# Over-the-wire lumen-apposing metal stent for the management of a chronic fistula after one-anastomosis gastric bypass

**DOI:** 10.1055/a-2552-0261

**Published:** 2025-03-21

**Authors:** Vincenzo Bove, Loredana Gualtieri, Maria Valeria Matteo, Martina De Siena, Valerio Pontecorvi, Cristiano Spada, Ivo Boskoski

**Affiliations:** 1 18654 Digestive Endoscopy Unit, Fondazione Policlinico Universitario Agostino Gemelli IRCCS, Rome, Italy

Gastro-gastric fistulas are uncommon complications of bariatric bypass surgery, but their management can be challenging 1 2 3 . We report a case of chronic gastro-gastric fistula after one-anastomosis gastric bypass (OAGB) treated with a lumen-apposing metal stent (LAMS), with the aim of presenting a viable treatment solution to the initial problem.

A 52-year-old woman presented with a chronic fistula after OAGB. The index procedure had been performed in another hospital and had been complicated by sepsis and pouch staple line leakage. The patient underwent surgical drainage, while the endoscopic examination showed an 8-mm orifice. An esophagogastric self-expandable metal stent had been placed, which migrated distally on day 10 and was then removed. Surprisingly, no further attempts were made to close the fistula.

A few months after surgery, the patient presented to our care; she was on parenteral nutrition and still had surgical drains in place. An abdominal computed tomography (CT) scan with Gastrografin and an upper gastrointestinal endoscopy showed a chronic fistula, with leakage into the peritoneal cavity between the gastric pouch and the remnant. As a result, a lumen-apposing metal stent (LAMS; 15 × 10-mm Hot-Axios; Boston Scientific, Massachusetts, USA) was placed between the gastric pouch and gastric remnant to close the leakage ( Video 1 ). No complications occurred. A follow-up CT scan and a gastric transit with Gastrografin showed complete closure of the leakage and rechanneling of the two structures. The patient was commenced on oral food intake and was discharged the day after the procedure. The LAMS was removed after 1 month.

An over-the-wire lumen-apposing metal stent is placed for the management of a chronic fistula after one-anastomosis gastric bypass.Video 1

At the 1-year follow-up, an endoscopic evaluation was performed, which revealed the persistence of the gastro-gastric fistula, with no evidence of leakage. The patient's weight remained stable, and it was decided that suturing of the gastro-gastric fistula was unnecessary, as it was no longer clinically relevant. The patient remains in excellent condition, with her weight stable 2 years after the procedure.

Endoscopy_UCTN_Code_TTT_1AO_2AI

## Notes

Endoscopy E-Videos https://eref.thieme.de/e-videos
*E-Videos* is an open access online section of the journal *Endoscopy* , reporting on interesting cases and new techniques in gastroenterological endoscopy. All papers include a high-quality video and are published with a Creative Commons CC-BY license. Endoscopy E-Videos qualify for HINARI discounts and waivers and eligibility is automatically checked during the submission process. We grant 100% waivers to articles whose corresponding authors are based in Group A countries and 50% waivers to those who are based in Group B countries as classified by Research4Life (see: https://www.research4life.org/access/eligibility/ ). This section has its own submission website at https://mc.manuscriptcentral.com/e-videos .

## Notes

Endoscopy E-Videos https://eref.thieme.de/e-videos
*E-Videos* is an open access online section of the journal *Endoscopy* , reporting on interesting cases and new techniques in gastroenterological endoscopy. All papers include a high-quality video and are published with a Creative Commons CC-BY license. Endoscopy E-Videos qualify for HINARI discounts and waivers and eligibility is automatically checked during the submission process. We grant 100% waivers to articles whose corresponding authors are based in Group A countries and 50% waivers to those who are based in Group B countries as classified by Research4Life (see: https://www.research4life.org/access/eligibility/ ). This section has its own submission website at https://mc.manuscriptcentral.com/e-videos .
